# Correction: Ormiston et al. Estrogen Signaling During Abrupt Involution and Long-Term Metabolic Signature Similar to Estrogen Receptor-Negative Breast Cancer. *Int. J. Mol. Sci.* 2026, 27, 1951

**DOI:** 10.3390/ijms27177558

**Published:** 2026-08-24

**Authors:** Kate Ormiston, Neelam Shinde, Gautam Sarathy, Allen Zhang, Morgan Bauer, Rajni Kant Shukla, Sara Alsammerai, Annapurna Gupta, Djawed Bennouna, Julia Wesolowski, Xiaoli Zhang, Rachel E. Kopec, Eswar Shankar, Kristin I. Stanford, Ramesh K. Ganju, Sarmila Majumder, Bhuvaneswari Ramaswamy, Daniel G. Stover

**Affiliations:** 1 Division of Medical Oncology, The Ohio State University Medical Center, Columbus, OH 43210, USA; 2 Human Nutrition Program, Department of Human Sciences, The Ohio State University, Columbus, OH 43210, USA; 3 College of Nursing, University of South Florida, Tampa, FL 33620, USA; 4 Dorothy M. Davis Heart and Lung Research Institute, Department of Surgery, Division of General and Gastrointestinal Surgery, The Ohio State University Wexner Medical Center, Columbus, OH 43210, USA; 5 Department of Pathology, The Ohio State University Wexner Medical Center, Columbus, OH 43210, USA

In the published publication [1], there was an error regarding the affiliations for Dr. Djawed Bennouna. The original affiliations listed were The Ohio State University and National Institute of Health (NIH). The updated affiliation should include only The Ohio State University. The authors state that the scientific conclusions are unaffected. This correction was approved by the Academic Editor. The original publication has also been updated.

## References

[1] Ormiston K., Shinde N., Sarathy G., Zhang A., Bauer M., Shukla R.K., Alsammerai S., Gupta A., Bennouna D., Wesolowski J. (2026). Estrogen Signaling During Abrupt Involution and Long-Term Metabolic Signature Similar to Estrogen Receptor-Negative Breast Cancer. Int. J. Mol. Sci..

